# What Dimensions of Risk Perception are Associated with Avoidance of Buying Processed Foods with Warning Labels?

**DOI:** 10.3390/nu12102987

**Published:** 2020-09-29

**Authors:** Cristian Adasme-Berríos, Luis Aliaga-Ortega, Berta Schnettler, Mercedes Sánchez, Consuelo Pinochet, Germán Lobos

**Affiliations:** 1 Department of Economy and Administration, Universidad Católica del Maule, 3460000 Talca, Chile; laliaga@ucm.cl (L.A.-O.); consuelo.pinochet@alu.ucm.cl (C.P.); 2 Faculty of Agricultural and Forest Sciences, Sci & Technol Bioresource Nucl BIOREN UFRO, Universidad de La Frontera, 4780000 Temuco, Chile; berta.schnettler@ufrontera.cl; 3 Department of Business Administration, IS-FOOD Research Institute, Public University of Navarra, 31006 Pamplona, Spain; mersan@unavarra.es; 4 Faculty of Economy and Business, Universidad de Talca, 3460000 Talca, Chile; globos@utalca.cl

**Keywords:** risk perception, nutritional warning labels, front-of-package, processed foods, nutrition information

## Abstract

Nutritional Warning Labels (NWLs) inform consumers about processed foods that exceed critical nutrient levels activating the risk perception in consumers. However, this information is limited. The objective was to identify the dimensions of risk perception and to study their associations with avoidance of buying processed foods with warning labels. A survey was applied to 807 decision-makers who choose what to eat at home. The four dimensions of risk perception (performance, financial, physical, and psychological) were identified through exploratory factor analysis. Through a multiple regression model, we determined the dimensions of risk perception and sociodemographic variables that affect the intention to avoid buying processed foods with NWLs. The results show that the performance risk, physical risk, and psychological risk positively affect the intention of avoiding processed foods with NWLs. In addition, the female gender considers NWLs to purchase foods. Nevertheless, the high frequency of consumption and belonging to a lower-income socioeconomic group are barriers to the use of NWLs. In conclusion, NWLs help people to choose processed food that does not impact negatively their food expectations, as well as their mental and physical health. However, health authorities must invest in nutrition education. Specifically, in groups who pay less attention to NWLs. Such groups include people with high daily consumption of processed foods, males, and low-income socioeconomic groups.

## 1. Introduction

The non-communicable diseases (NCDs) associated with obesity still are a challenge around the world [1]. One of the factors related to NCDs is the growing consumption of processed foods with high fat, sodium, sugar, and energy dense contents [2,3,4]. With the idea to reduce the consumption of processed foods, policymakers have proposed different health policies. Some of this health policy includes taxes and subsidies, increasing physical activities, reformulations of food products, and improvements in nutrition labeling [5,6].

In recent years, the presence of front-of-package labels as a nutrition label (FOP) on food has proliferated, either due to regulatory conditions in each country or due to marketing strategies. Different studies have analyzed the importance of FOP in the decision-making power of consumers. These studies showed that FOP in processed foods may alter the perception, evaluation, and dietary choices of individuals [7,8,9,10], even when people are pressured by the time [11]. Therefore, they constitute a public health tool that informs people through symbols and stimulates the choice of healthy products [9,12]. FOP can be into two categories [13]: (a) nutrient-specific labels (guideline daily amounts, traffic lights and Nutrition Warning Labels (NWLs)) and (b) summary labels (green keyhole schemes and graded indicators such as nutria-score or the Australian health star system). In this study, we will focus on NWLs, which are the most recent regulation and have attracted worldwide attention. This regulation, created in Chile, forces food companies to label their products with the NWL in the shape of a black octagon that explicitly indicates the excessive content of sugar, saturated fat, sodium, and/or calories concerning to a defined criterion. In this way, the population is informed about products that exceed the levels of critical nutrients associated with NCDs [14]. NWLs encourage consumers to make healthier eating decisions [15,16,17], given that the presence or number of NWLs modifies the consumer’s purchase preference in the short term [18]. It means that consumers prefer foods with fewer NWLs, because they perceive a risk to their health in products with these labels. This is based on mental models and psychological mechanisms that judge risk [19]. With regard to the risk perception, the “textual warning labels on soda” enhanced the perception of risk of contracting diabetes [20]. In other words, NWLs activate risk perception in consumers of processed foods. However, risk perception goes beyond a univariate concept that influences consumer decision-making. Different studies showed that the concept of risk perception is multidimensional because it captures affective, probabilistic and consequences dimension of risk [21]. However, there is only limited information about the dimensions of risk perception that explain the decision to avoid processed food with NWLs. In that sense, this research will provide evidence of a better understanding of the factors of risk perception associated with the NWLs that are needed in order to improve the effectiveness of this policy. 

### Theoretical Background and Literature Review

From a theoretical perspective, the NWL has convincing abilities based on the persuasive communication theories and principles of social and behavioral sciences [22]. In that context, the warning message conveyed by this type of label has a familiarity to people (similar to the transit stop sign), which helps the interpretation of nutrition information and then encourages the consumers to make a healthy food choice [23,24,25,26]. This means that NWLs capture the attention and it is understood by the consumer. Activating the risk perception in addition triggers behavioral intention and behavior change [22]. This model helps to explain, in conceptual terms, why consumers prefer foods with fewer NWLs. Placing the risk perception is a central plank in the final decision to purchase processed food with NWLs. However, scientific literature has not deepened in the components of risk perception associated with processed foods with warning labels. 

The concept of perceived risk refers to individual perceptions of uncertainty and the possible negative consequences of a specific event or behavior. The magnitude of the risk perception is determined by the probability of the risk occurring and its potential loss [27]. This loss, according to previous studies, is represented by five dimensions: performance, financial, physical, psychological, and social risk [28,29,30]. However, the dimensions of risk perception can vary according to the type of product or service [31]. Due to the independent nature of Chilean consumers’ decision-making and considering that the food choice is individual decision-making, the social risk is excluded from this study [32,33], and therefore researchers define the perceived risk based on a specific research context. In this study, perceived risk refers to how consumers perceive the possible losses that could occur due to the uncertainty generated by buying processed foods with warning labels. The dimensions of the perceived risk for this study are detailed below.

Performance risk refers to the fact that a product does not meet consumer expectations in terms of taste, nutrition, and/or price-quality ratio [34]. For example, products with NWLs present nutritional values that exceed the critical levels, adversely affecting what the consumer expected from the product. Financial risk is defined as the potential loss of money, resulting from the purchase of a product. In this context, some consumers can activate the perception of financial risk; due to food with NWLs being not worth the money spent. However, as NWLs are credibility attributes, they allow correcting information asymmetries [35], transforming the credibility attribute into a search attribute that guides the consumer to search for food without NWLs and therefore mitigate the financial risk. The physical risk corresponds to the probability that the acquisition of a food product becomes a threat to human life [36]. For example, the excessive consumption of critical nutrients such as saturated fats, carbohydrates, sodium and energy intake is associated with NCDs as a physical risk to health. To mitigate this physical risk, the NWLs encourage consumers to make healthier eating decisions [14,15,16,17]. The psychological risk is related to the loss of self-esteem and self-image due to not making a better decision when purchasing a product [37]. For example, NWLs can mitigate the psychological risk, since they facilitate the identification of processed foods with unfavorable nutrients [24]. That is, consumers may experience a loss of self-esteem from purchasing processed foods with NWLs to the point that they prefer reformulated processed products that ideally do not have NWLs or have the least amount of them [15,18]. 

Based on the previous background, the aims of this study are to identify the dimensions of risk perception and to study their associations with avoidance of buying processed foods with warning labels. We have hypothesized that the dimensions of performance risk, financial risk, physical risk, psychological risk, women in charge of food at home and people with high and middle socioeconomic status, would be associated with the avoidance buying processed foods with warning labels. These results will contribute to evidence about the effectiveness of NWLs, given that they activate risk perception in consumers, helping them to discriminate between healthy and less-healthy processed foods.

## 2. Materials and Methods 

### 2.1. Sample and Procedure

The study design was descriptive and cross-sectional, and was conducted in the city of Talca, Maule Region, Chile. The sample was obtained through convenience sampling and a questionnaire was applied to 807 decision-makers in charge of buying processed food for their home, who were of legal age (>18 years old). The survey data were collected through interviews conducted in public places near banks, stores, and supermarkets. The interviews were carried out from July to November 2018. Before data collection, the questionnaire was previously validated through a preliminary test of 10% of the sample, approaching the participants with the same method used in the final questionnaire. The problems detected were corrected for the final version of the questionnaire and the interview procedure. All subjects gave their informed consent for inclusion before they participated in the study. The study was conducted in accordance with the Declaration of Helsinki, and the protocol was approved by the Ethics Committee of Universidad Católica del Maule (Acta N° 85/2017).

### 2.2. Questionnaire

The questionnaire was divided into three sections. The first section compiled general information about the consumption of processed foods and included the frequency of daily consumption and place of purchase of processed foods. The second section included the risk perception scale adapted from previous studies [29,38]. This scale is generated by four dimensions that include: performance risk (PR), financial risk (FR), physical risk (PhysR), and psychological risk (PsyR). Each dimension is a compound of three items. Participants were asked to indicate the degree of agreement for each item using a seven-level Likert-type scale (1 = Totally disagree to 7 = Totally agree) regarding the perception of risk they felt in relation to the purchase of processed foods with NWLs. In addition, a scale about intention to avoid buying processed foods with NWLs was included (adapted from [39]). This scale was formulated of four items that included statements such as: (a) I try to avoid consuming food with NWLs; (b) I will suggest to my family members not to consume food with NWLs; (c) I will look for information to avoid buying food with NWLs and (d) I will avoid buying food with NWLs. Respondents were asked to indicate the degree of agreement for each item using a seven-level Likert-type scale (1 = strongly disagree to 7 = strongly agree). In this study, the Spanish version of the risk perception scale was used, which showed internal consistency with a Cronbach’s α coefficient between 0.76 and 0.89 [38]. For the scale that measured the intention to avoid buying processed foods with NWLs, internal consistency was also obtained, with a Cronbach’s α of 0.91 [39]. The third section of the questionnaire assessed sociodemographic variables such as gender, socioeconomic group, family group size, age, and the physiological variable of body mass index (BMI) composed of the ratio between weight (Kg) and height (m) squared. 

### 2.3. Statistical Analysis

The statistical analysis included two phases; the first included the descriptive statistics of the consumption of processed foods, sociodemographic variables, and the physiological variable of BMI. The second phase included multivariate models and was carried out in two stages. The first stage consisted of identifying the four dimensions of risk perception through an exploratory factor analysis of principal components with varimax rotation. Through this same technique, the intention to avoid buying processed foods with NWLs (IAB) was identified as a dimension. Once each of the dimensions had been identified, the second stage was carried out, in which a series of linear regressions were applied that increasingly added to the control variables to evaluate the estimated parameters. In this way, it was possible to quantify the relationship between the dependent variable (IAB) and the set of independent variables, which covered the four dimensions of risk perception (PR, FR, PhysR, PsyR) and sociodemographic and physiological elements. In the first regression, the dimensions of risk were controlled (stage 1). In the subsequent regressions (stage 2), the variables gender (female = 1; male = 0) and socioeconomic group (SEG1 = low; SEG2 = middle; SEG3 = high) were added as dummy variables; while the frequency of consumption of processed foods (CFPF) was added as a continuous variable, since it is measured as the average of processed food eaten per day. The sociodemographic and physiological variables in Table 1 were not considered as these were unable to explain the IAB in the model.

## 3. Results

### 3.1. Sample Characterization

Most of the decision-makers in charge of buying food for their home were women with an average age corresponded of 37 years. Regarding the physiological characteristics, an average weight of 71 kg was found, with an average height of 1.64 m and an average BMI of 26.04, which indicated overweight in the sample. More than half of the respondents declared living in a home consisting of three to four members. The low socioeconomic status (SEG1) represents 52.17% of the sample. In this group, the head of household maximum education level was secondary; their jobs were not specialized and their incomes lower than US$1050; middle socioeconomic status (SEG2) made up 42.38% of the sample. In this group, the head of household education level was university and they worked as professionals. Monthly income ranged between US$1051–US$3700; and only 5.45% of the respondents belonged to the high-income group (SEG3), which include professionals with postgraduate and top executives. The income is higher than US$3700.

Most interviewees (close to 90%) purchased processed foods at the supermarket. The second most selected option was neighborhood stores with 8.8% of preferences. In relation to frequency of consumption, the number of serves eaten of processed food per day for each interviewee were on average 2.67 unities (see Table 1).

### 3.2. Dimension of Risk Perception

To identify the dimensions of risk perception and the IAB dimension, two exploratory factor analyses were performed (see Table 2). The first analysis corresponded to the risk perception dimensions, which were extracted using principal components with varimax rotation. The 12 items were grouped to conform to four dimensions of perceived risk according to the scale. These four dimensions explained 83.15% of data variance. The internal consistency of each of the factors presented a Cronbach’s α > 0.85, and the KMO (Kaiser-Meyer-Olkin) was 0.879, which indicates a good fit of the data to the factor model. Bartlett’s test was significant. The second exploratory factor analysis was performed for the IAB dimension. Here, the four measured items revealed a factor that explained 70.63% of the variance. Cronbach’s α for this factor was 0.86 and the KMO was 0.813, with a significant Bartlett test, which also indicates a good fit of the data to the factor model.

Once the factor model was determined, we calculated the correlations of the evaluated factors. Table 3 shows that IAB correlated moderately with the factors that compound the perceived risk. However, the factor performance risk, financial risk, physical risk, and psychological risk showed a moderate to high correlation between them.

### 3.3. Determinants of the Intention to Avoid Buying Processed Foods with NWL

To identify the determinants of the intention to avoid buying processed foods with NWLs (IAB), a multiple linear regression model was carried out (see Table 4). Collinearity analysis was conducted on the regression models. In all cases the Variance Inflation Factor (VIF) was not greater than 1.78, indicating no serious multi-collinearity. In the first stage, only the risk factors and their incidence in the IAB were considered, because the perceived risk can impact consumption intention. In this first stage, it was found that the dimensions of performance risk, physical risk, and psychological risk contributed to IAB, with performance risk being the dimension that accounted for the most variance in the model. It indicates that an increase in the performance risk dimension causes the IAB to increase by 0.186 points. Physical risk had a similar effect, with 0.181 points, reflecting a positive increase in the willingness to discard processed food with NWLs. Ultimately, psychological risk-weighted 0.133, an effect that goes in the same direction as the previous dimensions. These three identified risks, increased the possibility of avoiding processed food with NWLs. On the other hand, the R-square statistic, which indicates the explanatory capacity of the model, reached 0.205. This result indicates that more than 20% of buyers’ consumption intentions is explained by the variation of risk factors.

Sociodemographic and physiological variables were added in the second model, maintaining the set of risk factors. Of these variables, gender, frequency of consumption of processed foods, and the socioeconomic group were able to explain the consumers’ intentions regarding processed foods with NWLs. The other variables that did not have a significant explanatory effect (age, family group size, place of purchase, and BMI) were discarded from the analysis. Regarding the factors that determined the model, performance risk continued to contribute positively with the highest weight, with an estimated beta coefficient of 0.194 points, therefore presenting an increase compared to the initial model. Regarding physical risk, the results showed a positive association with the dependent variable, with an estimated beta coefficient of 0.156 points, presenting a decrease compared to the initial model. Regarding psychological risk, this continued to be a significant factor, with an estimated beta coefficient of 0.135 points. Consequently, it was found that performance, physical, and psychological risk factors were the ones that contributed to increasing the IAB in consumers. Regarding the sociodemographic determinants, it was observed that when the food decision-maker was a woman, the IAB increased by 0.389 points. Conversely, belonging to a low-income socioeconomic group had a negative effect on the IAB, indicating that low-income people are less likely to avoid purchasing processed foods with NWLs. The frequency of consumption of processed foods also negatively affected the IAB by 0.073 points, which implies that the higher the frequency of consumption of processed foods, the more difficult it is to avoid consuming foods with NWLs. On the other hand, the R-square statistic of the second model increased the explanatory capacity, which reached 0.222. These statistics indicate than more than 20% of buyers’ consumption decision is explained by the variation in risk factors and the sociodemographic variables and frequency of consumption.

## 4. Discussion

This study used the theoretical model of risk perception, previously validated in other food topics [28]. As postulated in the hypothesis presented in this research, the findings at the aggregate level indicate that the presence of NWLs increases the perception of risk in people to processed foods. However, a disparate effect was obtained for the sociodemographic variables analyzed. 

### 4.1. Effect of NWLs on Risk Perception

Of the four dimensions of risk perception, only three of them were associated with the intention to avoid purchasing processed foods with NWLs. Performance risk was the most significant predictor of the intention to avoid eating processed foods with NWLs. It means that the presence of NWLs reduces the consumer’s perceptions of processed foods meeting their expectations. This finding is in line with previous studies, in which the authors showed that activation of performance risk is due to the lack of fulfillment of expectations, given that the analyzed foods present low nutritional contribution, low price-quality ratio, or flavors or textures that are not what the consumer expects [40,41]. As a way to improve customer expectations, producers of processed food should make an effort to communicate the positive attributes of products, aiming to reduce information asymmetries between food companies and final consumers. 

The second significant predictor for avoiding consumption of processed foods with NWLs was a physical risk. In this context, if the NWLs indicate a high content of critical nutrients such as sodium, sugars, saturated fats, and/or calories, people will consider that this food may be harmful to their health and that of others, so they will try to avoid it. This finding is in line with a previous study about the sodium NWL, in which the authors concluded that the inclusion of sodium warnings can discourage the choice of products with high content of this nutrient [42]. Therefore, producers of processed foods should use technology to produce foods that stand out for their nutritional properties and organoleptic characteristics, and that ideally do not negatively impact consumers’ health.

The third significant predictor was a psychological risk. The product with NWLs can generate some degree of dissatisfaction or disappointment in the consumer for not making a good decision about food. This disagreement can psychologically affect consumer self-esteem, both in men and women [43]. Therefore, it is imperative that food-producing companies must reformulate their products so that consumers have access to less harmful food for their physical and mental health.

### 4.2. Sociodemographic Aspects and their Relationship with NWLs

Furthermore, and unsurprisingly, women try to avoid processed foods with NWLs. This finding is in line with previous research showing that women are more aware of paying attention to credibility attributes such as NWLs [44,45]. Besides, processed foods create habits, which facilitate their consumption in adults [46]. According to these authors, this is due to the taste, children’s preference, convenience, addiction, and cost; therefore, these types of variables can act as a barrier to the effect of NWLs. Our results are consistent with this research as the frequency of daily consumption of processed foods also acts as a barrier to NWLs in some consumers. Although its impact is modest, but statistically significant, the consumption of processed foods is an ingrained custom in the population that is difficult to modify in the short term unless health authorities carry out promotional campaigns, which should be evaluated in time to measure their effectiveness.

Furthermore, our findings show that the lowest-income socioeconomic group is the one that least avoids the purchase of food with NWLs, becoming a risk group that can develop NCDs over time. This fact makes it necessary to intervene in this group through public policies. Previous studies have shown that this socioeconomic group also presents barriers to a healthy diet, which are represented by the low income it perceives, the need to satisfy hunger, the low level of schooling, and obesity [47,48].

### 4.3. NWL as An Attribute of Credibility in Consumers

The results of this study provide evidence that an NWL is a credibility attribute that corrects information asymmetries between food-producing companies and consumers. The credibility of NWLs “wake up” the risk perception of the consumers. In that sense, it is necessary to highlight that NWLs are mandatory (i.e., appear on all foods that have an objectively determined ‘unhealthy’ nutritional profile) to help consumers clearly distinguish between healthy and unhealthy products. Recent studies have shown that foods with a big number of NWLs will provide less satisfaction to consumers, from the view of the classical economic theory; it can generate less demand for the product [18,49]. Therefore, reformulated products without NWLs have a big chance of being purchased [15,17]. This chance is because the NWL acts as a brake on the appetizing signals of processed foods, as the NWL message increases people’s visual attention and allows an informed choice of food, focusing on improving diet quality and health status [50,51,52]. However, there are consumers for whom NWLs have little effect on their food choices [41], such as the male gender and the low-income socioeconomic group. Therefore, it is necessary to direct public policies towards this sector of the population to improve their eating habits. 

### 4.4. Study Limitations

Among the limitations presented by this study is the use of a non-probability type sampling, which makes the results not generalizable, as the sample is not representative of the socioeconomic situation in Chile. However, the study was carried out with the decision-makers who choose what to eat at home and who influence the eating habits of the family members. Another limitation of this research is that the study contains correlational (cross-sectional) data; therefore, the direction of causality cannot be established. Another limiting element of this study is that it did not consider product categories. For this reason, it is suggested to carry out further research for differentiated products, specifically by the number and type of NWL, to identify which dimensions of risk perception are more relevant in specific foods that exceed the levels of critical nutrients and have great demand in the market. In addition, we considered that future studies should develop specific items for social risk dimension to supplement the scale presented in this research.

## 5. Conclusions

Results from this research contribute to evidence about the effectiveness of warning labels, given that they partly activate different dimensions of the risk perception in consumers, helping them discriminate between healthy and less-healthy processed foods. This NWL allows people to discriminate between products that can affect their food expectations as well as their mental and physical health. Therefore, it is important that food-producing companies must transform their production processes to achieve foods that comply with these types of standards, and that generate less adverse effects on the population. Additionally, health authorities must make a real effort to provide nutritional education to those groups that place less importance on warning labels. Such groups include people´s high daily consumption of processed foods, the group of males and low-income socioeconomic groups. In this way, consumers will be able to make informed choices about the types of processed foods that they consume.

## Figures and Tables

**Table 1:** Descriptive Statistics for Sociodemographic and Physiological Variables.

Categorical Variables		Frequency	Percentage
Gender	Male	292	36.18
	Female	515	63.82
Socioeconomic status	Low (SEG1)	421	52.17
	Middle (SEG2)	342	42.38
	High (SEG3)	44	5.45
Family group size	1–2 members	165	20.50
	3–4 members	445	55.10
	5 or more	197	24.40
Shop place	Supermarket	720	89.22
	Neighborhood stores	71	8.80
	Casinos/Cafeteria	10	1.24
	Kiosks	3	0.37
	Street trade	3	0.37
Other Variables		Mean.	Sd.
Age		37.31	13.95
Height (m)		1.64	0.08
Weight (Kg)		71.04	14.19
BMI		26.04	4.35
Daily Consumption Frequency of Processed Foods (CFPF)		2.67	1.28

**Table 2:** Exploratory factor analysis for the dimensions of perceived risk and intention to avoid buying processed foods with nutrition warning labels (NWLs) and descriptive statistic of the items (*n* = 807).

Items			Factor Loadings			
	Means	Std. D.	Physical Risk (PhysR)	Performance Risk (PR)	Financial Risk (FR)	Psychological Risk (PsyR)
PhysR3. You are concerned about the physical damage associated with its consumption	5.48	1.91	0.878	0.160	0.142	0.170
PhysR2. You consider that its consumption can be harmful to your health	5.52	1.89	0.875	0.134	0.149	0.158
PhysR1. You are concerned about the side effects it may cause you or a family member	5.33	2.02	0.857	0.213	0.111	0.157
PR3. You fear that the product will not meet your needs	4.31	2.33	0.119	0.870	0.229	0.186
PR2. You fear that it may not provide you benefits	4.16	2.32	0.196	0.847	0.253	0.223
PR1. You are concerned that it is not a safe and reliable food	4.52	2.33	0.273	0.813	0.247	0.192
FR3. You are concerned that the purchase of this food is not worth the money spent	3.94	2.32	0.110	0.237	0.864	0.220
FR2. You are concerned that it is not a good acquisition because it is more expensive than other available brands	3.75	2.28	0.133	0.252	0.850	0.218
FR1. You think it is not a good way to spend your money.	3.97	2.34	0.215	0.234	0.775	0.280
PsyR2. You are worried because of doubts about whether you have been right with your decision	3.54	2.23	0.122	0.229	0.233	0.858
PsyR1. You are worried when buying these products	3.47	2.27	0.156	0.171	0.235	0.846
PsyR3. You consider that you have not been careful when buying processed foods with NWLs	4.10	2.31	0.254	0.176	0.213	0.778
Cronbach α			0.89	0.91	0.89	0.88
KMO			0.879			
Bartlett Test			0.000			
			**Factor Loadings**			
**Items**	**Means**	**Std. D.**	**Intention to Avoid Buying Processed Foods with NWL (IAB)**			
IAB4. You will avoid buying food with NWLs	5.06	2.03		0.881		
IAB3. You will seek information to avoid buying food with NWLs	4.73	2.14		0.847		
IAB2. You will suggest to my family members not to consume food with NWLs	5.14	2.08		0.830		
IAB1. You try to avoid consuming food with NWLs	4.76	2.19		0.802		
**Cronbach α**				0.86		
**KMO**				0.813		
**Bartlett Test**				0.000		

**Table 3:** Bivariate correlations of the perceived risk dimensions (*n* = 807).

	IAB		PR		FR		PhysR		PsyR
PR	0.387	**	1						
FR	0.292	**	0.576	**	1				
PhysR	0.350	**	0.450	**	0.390	**	1		
PsyR	0.347	**	0.505	**	0.563	**	0.429	**	1

** *p* < 0.01.

**Table 4:** Results of the multiple regression model performed on an intention to avoid buying processed foods with NWLs.

Predictor	Coef.	Std. Err.	t	R^2^	Adj. R^2^	F
Step 1:						
**Intercept**	2.622	0.187	14.044 ***			
**PR**	0.186	0.034	5.461 ***			
**FR**	0.005	0.005	0.130			
**PhysR**	0.181	0.037	4. 923 ***			
**PsyR**	0.133	0.035	3.779 ***	0.205	0.201	51.650 ***
Step 2:						
**Intercept**	2.878	0.239	12.046 ***			
**PR**	0.194	0.034	5.746 ***			
**FR**	0.003	0.035	0.085			
**PhysR**	0.156	0.037	4.237 ***			
**PsyR**	0.135	0.035	3.836 ***			
**Gen (Women)**	0.389	0.116	3.363 ***			
**CFPF**	−0.073	0.043	−1.673 **			
**SEG_1_**	−0.372	0.115	−3.226 ***			
**SEG_2_**	Omitted					
**SEG_3_**	−0.200	0.252	−0.803	0.230	0.222	29.826 ***

** *p* < 0.05; *** *p* < 0.01.
